# Golgi Protein 73 and Laminin‐γ2 Monomer‐Based Score for the Diagnosis of Advanced Liver Fibrosis in Patients With Metabolic Dysfunction‐Associated Steatotic Liver Disease

**DOI:** 10.1002/jgh3.70455

**Published:** 2026-08-02

**Authors:** Xin‐Tong Ng, Jin‐Ying Teh, Pavai Sthaneshwar, Izzatul Aliaa Badaruddin, Pavitratha Puspanathan, Nik Raihan Nik Mustapha, Sanjiv Mahadeva, Wah‐Kheong Chan

**Affiliations:** ^1^ Faculty of Medicine, Gastroenterology and Hepatology Unit, Department of Medicine University of Malaya Kuala Lumpur Malaysia; ^2^ Faculty of Medicine, Clinical Diagnostic Laboratory, Department of Pathology University of Malaya Kuala Lumpur Malaysia; ^3^ Faculty of Medicine, Medical Diagnostic Laboratory, Department of Pathology Universiti Kebangsaan Malaysia Bangi Selangor Malaysia; ^4^ Department of Pathology Hospital Pulau Pinang Pulau Pinang Malaysia; ^5^ Department of Pathology Hospital Sultanah Bahiyah Alor Setar Kedah Malaysia

**Keywords:** GLAS score, GP73, LG2m, MASLD

## Abstract

**Background:**

This study evaluated the diagnostic performance of Golgi protein 73 (GP73), laminin‐γ2 monomer (LG2m), and the GLAS score in detecting advanced fibrosis in patients with metabolic dysfunction‐associated steatotic liver disease (MASLD).

**Methods:**

This study included patients with MASLD who underwent liver biopsy. The performance of serum levels of GP73 and LG2m and the GLAS score to diagnose advanced fibrosis was evaluated using area under receiver operating characteristic curves (AUROC).

**Results:**

The data for 209 patients with MASLD were analyzed [median age 52 (42–58) years, 53.1% men, 71.3% had steatohepatitis, 18.1% had advanced fibrosis]. The serum levels of GP73 and LG2m and the GLAS score were significantly higher in patients with advanced fibrosis compared to patients without advanced fibrosis [GP73: 83 ± 50 ng/mL vs. 61 ± 35 ng/mL, *p* = 0.013; LG2m: 31 (14–48) pg/mL vs. 17 (12–23) pg/mL, *p* < 0.001; GLAS score: 0.89 (0.49–0.97) vs. 0.47 (0.15–0.78), *p* < 0.001]. The AUROC of serum levels of GP73 and LG2m and GLAS for diagnosing advanced fibrosis was 0.63 (0.52–0.74), 0.73 (0.62–0.83), and 0.72 (0.61–0.82), respectively. Using the optimal cut‐off of 27 pg/mL, the sensitivity, specificity, positive predictive value, and negative predictive value of LG2m for diagnosing advanced fibrosis were 60.5%, 86.0%, 48.9%, and 90.7%, respectively. Using the optimal cut‐off of 0.75, the corresponding values for the GLAS score were 65.8%, 73.7%, 35.7%, and 90.6%, respectively.

**Conclusion:**

Serum LG2m level and the GLAS score have fair accuracy for the diagnosis of advanced fibrosis in patients with MASLD.

## 1 Introduction

Metabolic dysfunction‐associated steatotic liver disease (MASLD) has emerged as the most common cause of chronic liver disease, with an estimated prevalence of around 38% worldwide [1]. The more severe form of the disease, metabolic dysfunction‐associated steatohepatitis (MASH), can progress to fibrosis, liver cirrhosis, and hepatocellular carcinoma (HCC) [2]. For patients with chronic liver disease, liver fibrosis is a strong predictor of the course of the disease and clinical outcomes [3]. Early intervention can help reverse fibrosis and even early cirrhosis. Therefore, effective methods for detecting and monitoring liver fibrosis are crucial. Liver biopsy has long been considered the gold standard for assessing liver fibrosis [4]. However, various limitations prohibit widespread use of liver biopsy, including invasiveness, potential complications, suboptimal specimen, sampling variability, and observer variability [5, 6, 7]. Consequently, alternative approaches that are simple, non‐invasive, and reliable for evaluating liver fibrosis are necessary [8].

Golgi protein 73 (GP73) is a resident Golgi transmembrane glycoprotein primarily expressed in biliary epithelial cells but rarely in hepatocytes in normal livers [9]. Serum GP73 concentration increases as a result of diseased or damaged cells releasing GP73 into the extracellular space [10]. Many studies have shown that GP73 may be a potential diagnostic marker for hepatocellular carcinoma (HCC) [11, 12, 13]. However, other studies suggest that GP73 may not be specific for diagnosing HCC, but may be a potential surrogate biomarker for liver fibrosis and cirrhosis [10, 14]. Emerging evidence supports the utility of serum GP73 as a fibrosis biomarker in chronic liver diseases such as chronic hepatitis B and C infection [15, 16], but the diagnostic value of GP73 in advanced fibrosis in patients with MASLD is less clear. Serum laminin‐γ2 monomer (LG2m) is a potential biomarker for HCC surveillance in patients with chronic hepatitis B and C infection [17, 18]. Recently, an algorithm combining GP73 and LG2m serum biomarkers with age and sex (GLAS) was reported to have high sensitivity and specificity in detecting fibrosis and liver cirrhosis across two large and diverse patient cohorts [19]. The main aim of our study was to evaluate the usefulness of LG2m, GP73 and the GLAS score for the diagnosis of advanced fibrosis in patients with MASLD. As exploratory analyses, we also compared them with the Fibrosis‐4 score (FIB‐4) and liver stiffness measurement (LSM) for the diagnosis of advanced fibrosis and explored their role in the two‐step approach for identifying patients with advanced fibrosis. We believe the GLAS score may have a role as a second line test in the two‐step approach for identifying patients with advanced fibrosis.

## 2 Methods

The study subjects were patients with non‐alcoholic fatty liver disease (NAFLD) who had undergone liver biopsy at the University of Malaya Medical Centre between 2012 and 2015. The diagnosis of NAFLD was based on ultrasonography finding of fatty liver and exclusion of significant alcohol intake, use of medications that can cause fatty liver, viral hepatitis B and C infection, and other causes of chronic liver disease where indicated [20]. All subjects fulfilled the criteria for MASLD, defined as hepatic steatosis with at least one cardiometabolic risk factor and without significant alcohol intake, which was defined as > 20 g per day in women and > 30 g per day in men [21], and the term MASLD was retrospectively assigned. Demographic, anthropometric and relevant clinical data were obtained using a standard protocol on the day of the liver biopsy procedure. Body mass index (BMI) was calculated by dividing weight in kilograms by the square of height in meters. Obesity was defined as BMI ≥ 25 kg per m^2^ [22]. Waist circumference was measured at the mid‐point between the lowest margin of the least palpable rib and the top of the iliac crest in the standing position. Central obesity was defined as waist circumference > 90 cm for men and > 80 cm for women [23]. Venous blood was drawn after an overnight fast on the day of the liver biopsy procedure for complete blood count, blood glucose, glycated hemoglobin (HbA1c), lipid profile, and liver profile. Additional blood samples were collected in a plain tube, processed to plasma and stored at −80°C until further analysis. Biochemical measurements were performed using standard laboratory procedures. Healthy controls were recruited from individuals attending the Endoscopy Unit, University of Malaya Medical Centre for investigation of dyspepsia or screening colonoscopy. All healthy controls had no known medical illness and had an ultrasound examination to exclude fatty liver. Venous blood was drawn after an overnight fast, collected in a plain tube, processed to plasma and stored at −80°C until further analysis. The healthy controls were included to provide data of LG2m, GP73 and the GLAS score in healthy persons without fatty liver and were not planned to be age‐ and gender‐matched with the MASLD patients in the study.

### 2.1 Liver Biopsy and Histological Assessment

Ultrasonography‐guided percutaneous liver biopsy was performed by either one of two experienced operators (WKC, SM) using an 18G Temno II semi‐automatic biopsy needle (Cardinal Health, Dublin, Ohio, USA). Liver biopsy specimens were processed using standard laboratory procedures. Liver biopsy slides were stained using hematoxylin and eosin, as well as Masson's trichrome. An experienced histopathologist (NRNM), blinded to the clinical data, examined the slides. Histopathological findings were reported according to the Non‐Alcoholic Steatohepatitis Clinical Research Network Scoring System [24]. MASH was defined as the presence of steatosis, lobular inflammation, and ballooning with or without fibrosis. Fibrosis stages 1a, 1b, and 1c were considered stage 1 for the purpose of analysis. Fibrosis stage of three or more was considered advanced fibrosis.

### 2.2 Measurement of Serum GP73 and LG2m Levels and the GLAS Score

Serum GP73 and LG2m concentrations were measured using research use only (RUO) assays on the automated Abbott Alinity *i* system (Abbott Laboratories, North Chicago, IL, USA) by a single operator (IAB), who was blinded to demographic, anthropometric, clinical and histological data. Both assays employ two‐step sandwich immunoassay methods with paramagnetic microparticles coated with monoclonal antibodies specific to GP73 or LG2m. The resulting chemiluminescent signal is directly proportional to the concentration of the respective analyte in human, enabling quantitative analysis. The within‐laboratory precision was < 3% and 4.6% for GP73 and LG2m, respectively. The lower limit of quantitation (LOQ) was 0.20 ng/mL for GP73 and 3.45 pg/mL for LG2m. The assays demonstrated a linear measuring range up to 1000 ng/mL for GP73 and 5000 pg/mL for LG2m. Measurements were performed according to the manufacturer's recommended procedures, and the assays fulfilled established criteria for both precision and analytical accuracy. The GLAS score was calculated using a proprietary formula consisting of four variables that is, GP73, LG2m, age and sex [19]. The proprietary formula was not known to the investigators and only the data on GP73, LG2m, age and sex were provided to Abbott Laboratories for calculation of the GLAS score.

### 2.3 Fibrosis‐4 Index

FIB‐4 was calculated using the following formula: age (years) × aspartate aminotransferase/[platelet (×10^9^/L) × square root (alanine aminotransferase)]. The cut‐off value of < 1.3 was used in patients who are < 65 years old, while the cut‐off value of < 2.0 was used in patients who are ≥ 65 years old, to exclude advanced fibrosis [25].

### 2.4 Liver Stiffness Measurement

LSM was performed after overnight fasting using the Fibroscan 502 Touch (EchoSens, Paris, France) on the same day as the liver biopsy procedure. Adequate pressure of the probe on the skin surface, good layering on TM mode and a straight imaginary line on A mode were ensured for each measurement. An examination was considered successful if there were 10 valid measurements, and reliable if the interquartile range (IQR)/median for LSM was ≤ 30% or the LSM was < 7.1 kPa when the IQR/median for LSM was > 30% [26]. Subjects with unsuccessful or unreliable examination were excluded from the analysis on LSM. For the diagnosis of advanced fibrosis according to LSM value, < 10 kPa indicated the absence of advanced fibrosis and ≥ 15 kPa indicated the presence of advanced fibrosis. An LSM value between 10 and 14.9 kPa was considered intermediate.

### 2.5 Statistical Analysis

Data was analyzed using a standard statistical software program (SPSS 30.0). Normality of each continuous variable was determined by Kolmogorov–Smirnov test. Variable was considered normally distributed if the *p*‐value was > 0.05. Continuous variables that were normally distributed were expressed as mean ± standard deviation and analyzed using *t*‐test or ANOVA test, as appropriate. Continuous variables that were not normally distributed were expressed as median (interquartile range) and analyzed using Mann–Whitney test or Kruskal‐Wallis test, as appropriate. Categorical variables were expressed as percentages and analyzed using chi‐square test or Fisher's exact test, as appropriate. Significance was assumed when *p* < 0.05. Boxplots were used to compare the serum levels of GP73 and LG2m and the GLAS score between fibrosis stages and between patients with and without advanced fibrosis. The performance of serum levels of GP73 and LG2m and the GLAS score for the diagnosis of advanced fibrosis was determined using area under receiver‐operating characteristics curve (AUROC). AUROC was interpreted as follows: 0.90–1.00 = excellent, 0.80–0.90 = good, 0.70–0.80 = fair, < 0.70 = poor. Optimal cut‐off values for serum levels of GP73 and LG2m and the GLAS score for the diagnosis of advanced fibrosis were the values that provided the greatest sum of sensitivity and specificity. The sensitivity, specificity, positive predictive value and negative predictive value using the optimal cut‐off values were determined.

## 3 Results

### 3.1 Patient Characteristics

The data for 209 patients with MASLD were analyzed. Patient characteristics are summarized in Table 1. The median age of the study population was 52 (42–58) years, with 53.1% of the participants being men. The median length of liver biopsy specimen was 15 (12–16) mm, and the number of portal tracts was 8 (6–10). The majority of patients (71.3%, 149/209) had MASH and 18.1% (38/209) had advanced fibrosis. The distribution of histological fibrosis stage was as follows: F0 (33.5%, 70/209), F1 (40.7%, 85/209), F2 (7.7%, 16/209), F3 (14.8%, 31/209), and F4 (3.3%, 7/209). The median age of the healthy controls was 24 (23–55) years, with 19.4% of the participants being men.

**TABLE 1:** Characteristics of patients according to fibrosis stages.

	F0, *n* = 70	F1, *n* = 85	F2, *n* = 16	F3, *n* = 31	F4, *n* = 7	Without advanced fibrosis, *n* = 171	With advanced fibrosis, *n* = 38	*p* *
Age, years	48.5 (39.8–54.0)	53.0 (38.5–61.0)	51.0 (35.3–56.3)	58.0 (50.0–62.0)	58.0 (51.0–64.0)	51.0 (39.0–57.0)	58.0 (50.8–62.0)	< 0.001
Male, %	54.3	52.9	68.8	45.2	42.9	55.0	44.7	0.253
Weight, kg	76.1 ± 13.4	78.3 ± 14.2	85.9 ± 12.7	79.3 ± 17.5	74.3 ± 9.1	78.1 ± 13.9	78.4 ± 16.3	0.907
Obesity, %	81.4	85.9	100.0	87.1	100.0	85.4	89.5	0.509
BMI, kg per m^2^	29.0 ± 4.2	30.2 ± 4.3	30.7 ± 3.9	30.2 ± 5.3	30.2 ± 3.6	29.6 (27.1–31.7)	29.4 (26.1–33.6)	0.766
Waist circumference, cm	97 ± 10	97 ± 9	103 ± 10	101 ± 12	100 ± 7	98 ± 10	101 ± 11	0.077
Central Obesity, %	90.0	95.3	100.0	100.0	100.0	93.6	100.0	0.108
Diabetes, %	30.0	57.6	43.8	87.1	85.7	45.0	86.8	< 0.001
Hypertension, %	48.6	57.6	56.3	87.1	71.4	53.8	84.2	< 0.001
Dyslipidemia, %	68.6	75.3	68.8	87.1	71.4	71.9	84.2	0.118
Ischemic heart disease, %	2.9	2.4	0.0	6.5	0.0	2.3	5.3	0.329
CAP, dB/m	311 ± 46	322 ± 40	330 ± 21	331 ± 57	296 ± 36	327 (296–346)	326 (290–363)	0.470
IQR for CAP, dB/m	7 (5–10)	7 (5–11)	7 (5–9)	6 (4–11)	6 (4–8)	7 (5–10)	6 (4–10)	0.121
E, kPa	5.9 (4.6–6.8)	7.8 (6.4–10.4)	9.4 (7.9–10.9)	13.9 (11.2–20.1)	20.2 (12.1–35.3)	6.9 (5.8–8.9)	14.8 (11.5–21.2)	< 0.001
IQR/Median for E, %	11.5 (7.3–18.0)	13.0 (10.0–17.0)	13.5 (10.3–20.8)	15.0 (9.0–21.0)	13.0 (10.0–14.0)	12.0 (9.0–17.3)	13.0 (9.0–18.8)	0.501
FBS, mmol/L	5.5 (4.9–6.0)	5.9 (5.0–7.1)	6.0 (5.1–7.3)	7.0 (5.9–8.7)	6.8 (6.2–7.1)	5.6 (5.0–6.8)	6.9 (6.0–8.5)	< 0.001
Serum Insulin level, mIU/L	18.6 (13.5–27.8)	24.2 (16.4–39.2)	27.3 (24.1–40.4)	31.2 (20.9–51.3)	37.4 (24.8–66.7)	20.9 (15.2–35.0)	31.5 (21.8–47.0)	< 0.001
HbA1C, %	5.8 (5.5–6.7)	6.2 (5.6–7.3)	6.0 (5.4–7.3)	7.1 (6.1–8.6)	6.5 (5.4–7.2)	6.0 (5.5–7.1)	7.1 (5.9–7.8)	0.002
Albumin, g/L	43 ± 4	43 ± 3	43 ± 3	42 ± 4	44 ± 4	43 (41–45)	42 (40–46)	0.152
Bilirubin, umol/L	10 (7–15)	11 (9–16)	10 (8–14)	10 (8–13)	16 (13–22)	10 (8–15)	11 (9–15)	0.274
ALT, IU/L	55 (41–77)	66 (42–108)	98 (72–121)	72 (44–127)	56 (44–101)	61 (42–99)	71 (44–111)	0.231
AST, IU/L	32 (25–39)	43 (28–59)	60 (30–71)	61 (40–83)	53 (36–72)	35 (27–52)	59 (39–79)	< 0.001
GGT, IU/L	49 (33–96)	71 (40–107)	98 (56–184)	109 (77–148)	123 (97–171)	66 (39–108)	114 (79–149)	< 0.001
Platelet, 10^9^/L	291 ± 52	276 ± 76	280 ± 53	242 ± 65	201 ± 59	280 (244–317)	226 (188–288)	< 0.001
Triglyceride, mmol/L	1.60 (1.20–2.00)	1.60 (1.30–2.10)	1.45 (1.05–1.85)	1.70 (1.30–2.00)	1.30 (0.90–2.10)	1.60 (1.29–2.00)	1.65 (1.25–2.00)	0.818
Total cholesterol, mmol/L	5.06 ± 0.88	4.98 ± 1.14	4.86 ± 1.33	4.75 ± 1.33	4.31 ± 0.77	4.85 (4.20–5.70)	4.40 (4.08–5.40)	0.076
HDL, mmol/L	1.17 (0.99–1.40)	1.13 (0.99–1.29)	1.06 (0.85–1.27)	1.18 (1.03–1.30)	1.26 (1.16–1.60)	1.13 (0.98–1.33)	1.20 (1.03–1.38)	0.228
LDL, mmol/L	3.12 ± 0.85	3.03 ± 1.03	3.08 ± 1.03	2.78 ± 1.15	2.31 ± 0.61	3.07 ± 0.96	2.70 ± 1.08	0.034

*Note:* Fibrosis stage of three or more was considered advanced fibrosis.

Abbreviations: ALT, alanine aminotransferase; AST, aspartate aminotransferase; BMI, body mass index; CAP, controlled attenuation parameter; E, liver stiffness measurement; FBS, fasting blood sugar; GGT, gamma‐glutamyl transferase; HbA1C, glycated hemoglobin; HDL, high‐density lipoprotein cholesterol; IQR, interquartile range; LDL, low‐density lipoprotein cholesterol.

* *p*‐value between groups, that is, with advanced fibrosis and without advanced fibrosis, were calculated using *t*‐test or Mann–Whitney test, where appropriate, for continuous variables, and chi‐square test or Fisher exact test, where appropriate, for categorical variables.

### 3.2 GP73, LG2m and GLAS According to Fibrosis Stages

**FIGURE 1:**
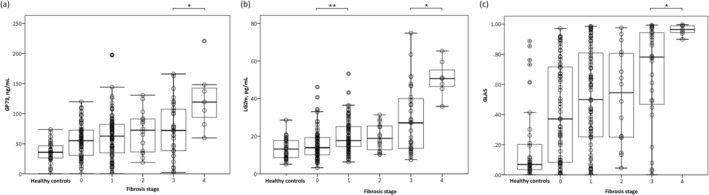
Boxplots showing the serum levels of (a) GP73, and (b) LG2m and (c) the GLAS score in healthy controls and patients with different fibrosis stages (*p < 0.05, **p < 0.001). Serum GP73 levels were significantly higher in patients with fibrosis stage F4 compared to those with fibrosis stage F3 (*p* = 0.013). Serum LG2m levels were significantly higher in patients with fibrosis stage F1 compared to those with fibrosis stage F0 (*p* < 0.001), and in patients with fibrosis stage F4 compared to those with fibrosis stage F3 (*p* = 0.004). The GLAS scores were significantly higher in patients with fibrosis stage F4 compared to those with fibrosis stage F3 (*p* = 0.005). GLAS, algorithm combining GP73 and LG2m with age and sex; GP73, Golgi protein 73; LG2m, laminin‐γ2 monomer.

The serum levels of GP73 and LG2m and the GLAS score according to fibrosis stages are shown in Figure 1a–c, respectively. The serum level of GP73 for fibrosis stages F0, F1, F2, F3 and F4 was 56 ± 28, 63 ± 39, 70 ± 34, 74 ± 46 and 125 ± 52 ng/mL, respectively (*p* < 0.001). Serum GP73 levels were significantly higher in patients with fibrosis stage F4 compared to those with fibrosis stage F3 (*p* = 0.013). However, there were no significant differences in GP73 levels between patients with fibrosis stage F0 and F1, F1 and F2, or F2 and F3. The serum level of LG2m for fibrosis stages F0, F1, F2, F3 and F4 was 14 (10–19) pg/mL, 18 (14–25) pg/mL, 19 (12–26) pg/mL, 27 (13–41) pg/mL and 51 (45–60) pg/mL, respectively (*p* < 0.001). Serum LG2m levels were significantly higher in patients with fibrosis stage F1 compared to those with fibrosis stage F0 (*p* < 0.001), and in patients with fibrosis stage F4 compared to those with fibrosis stage F3 (*p* = 0.004), but not significantly different between patients with fibrosis stage F1 and F2, or F2 and F3. The GLAS score for fibrosis stages F0, F1, F2, F3 and F4 was 0.37 (0.08–0.72), 0.50 (0.25–0.82), 0.55 (0.25–0.81), 0.78 (0.45–0.95) and 0.96 (0.94–0.99), respectively (*p* < 0.001). The GLAS scores were significantly higher in patients with fibrosis stage F4 compared to those with fibrosis stage F3 (*p* = 0.005), but not significantly different between patients with fibrosis stage F0 and F1, F1 and F2, or F2 and F3.

### 3.3 GP73, LG2m and GLAS in Patients With and Without Advanced Fibrosis

**FIGURE 2:**
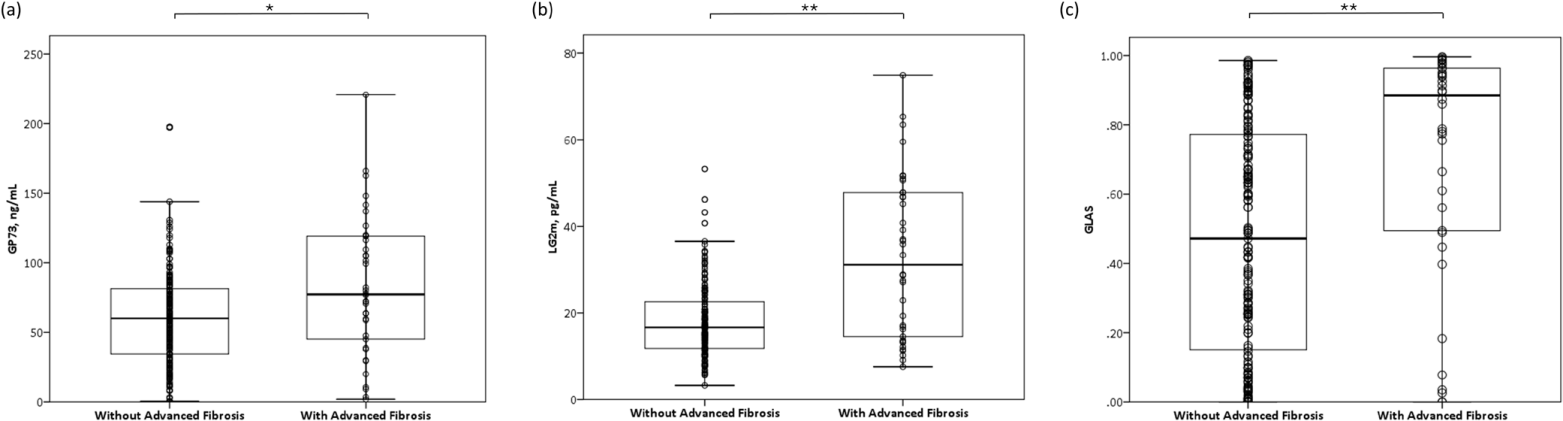
Boxplots showing the serum levels of (a) GP73, and (b) LG2m and (c) the GLAS score in patients with and without advanced fibrosis (*p < 0.05, **p < 0.001). The serum levels of GP73 and LG2m and the GLAS scores were significantly higher in patients with advanced fibrosis compared to those without advanced fibrosis (*p* = 0.013, *p* < 0.001, and *p* < 0.001, respectively). GLAS, algorithm combining GP73 and LG2m with age and sex; GP73, Golgi protein 73; LG2m, laminin‐γ2 monomer.

The serum levels of GP73 and LG2M and the GLAS score in patients with and without advanced fibrosis are shown in Figure 2a–c, respectively. The serum levels of GP73 were significantly higher in patients with advanced fibrosis compared to those without advanced fibrosis (83 ± 50 vs. 61 ± 35 ng/mL, *p* = 0.013). Similarly, serum levels of LG2m were significantly higher in patients with advanced fibrosis compared to those without [31 (14–48) vs. 17 (12–23) pg/mL, *p* < 0.001]. In addition, the GLAS score was significantly higher in patients with advanced fibrosis compared to those without [0.89 (0.49–0.97) vs. 0.47 (0.15–0.78), *p* < 0.001].

### 3.4 GP73, LG2m and GLAS for the Diagnosis of Advanced Fibrosis

**FIGURE 3:**
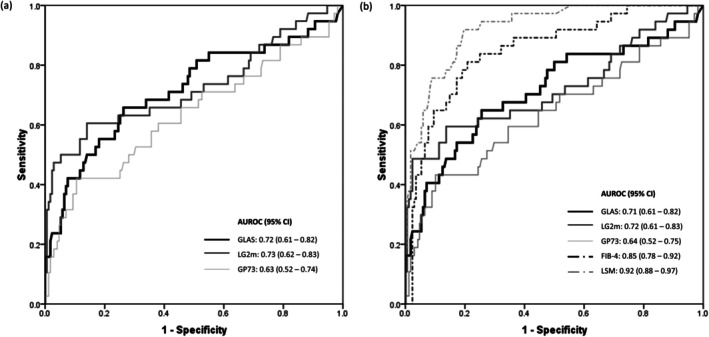
(a) AUROC of GP73, LG2m, and the GLAS score for the diagnosis of advanced fibrosis in the overall study population of 209 patients (b) AUROC of GP73, LG2m, the GLAS score, FIB‐4 and LSM for the diagnosis of advanced fibrosis in the 205 patients with complete data for the analysis. AUROC was interpreted as follows: 0.90–1.00 = excellent, 0.80–0.90 = good, 0.70–0.80 = fair, < 0.70 = poor. FIB‐4, Fibrosis‐4 index; GLAS, algorithm combining GP73 and LG2m with age and sex; GP73, Golgi protein 73; LG2m, laminin‐γ2 monomer; LSM, liver stiffness measurement.

The AUROC of serum GP73 and LG2m levels, the GLAS score and FIB‐4 for the diagnosis of advanced fibrosis is shown in Figure 3. The AUROC of serum GP73 levels for differentiating patients with advanced fibrosis from those without was 0.63 (95% CI: 0.52–0.74). Using the optimal cut‐off of 99 ng/mL, the sensitivity, specificity, positive predictive value and negative predictive value of serum GP73 levels were 42.1%, 89.5%, 47.1% and 87.4%, respectively. For serum LG2m levels, the AUROC was 0.73 (95% CI: 0.62–0.83), with the sensitivity, specificity, positive predictive value and negative predictive value of 60.5%, 86.0%, 48.9% and 90.7%, respectively, at the optimal cut‐off of 27 pg/mL. The GLAS score had an AUROC of 0.72 (95% CI: 0.61–0.82), with the sensitivity, specificity, positive predictive value and negative predictive value of 65.8%, 73.7%, 35.7% and 90.6%, respectively, at the optimal cut‐off of 0.75.

FIB‐4 demonstrated a higher AUROC of 0.85 (95% CI: 0.78–0.92), compared to the GLAS score. Using the optimal cut‐off of 1.2, the sensitivity, specificity, positive predictive value, and negative predictive value of FIB‐4 were 81.6%, 79.4%, 47.0%, and 95.1% respectively, higher than those of the GLAS score. As expected, LSM demonstrated the highest AUROC of 0.92 (95% CI: 0.88–0.97). Using the optimal cut‐off of 9.7, the sensitivity, specificity, positive predictive value, and negative predictive value of LSM for the diagnosis of advanced fibrosis were 91.9%, 80.4%, 50.7%, and 97.8% respectively. The AUROC of LG2m, GP73, the GLAS score, FIB‐4, and LSM for the diagnosis of advanced fibrosis in different subgroups stratified by age, diabetes status, and BMI is shown in Table 2. Similar to FIB‐4, the AUROC of the GLAS score for the diagnosis of advanced fibrosis appeared lower in patients ≥ 65 years and in patients with diabetes, while LSM appeared less accurate in patients ≥ 65 years but unaffected by diabetes status. The BMI status did not appear to affect the performance of the biomarkers, except for GP73, where the AUROC appeared lower in patients with BMI < 30 kg per m^2^. However, the results of these subgroup analyses should be interpreted cautiously due to the relatively small number of patients with advanced fibrosis within each subgroup.

**TABLE 2:** The AUROC (95% CI) of LG2m, GP73, the GLAS score, FIB‐4, and LSM for the diagnosis of advanced fibrosis in different subgroups stratified by age, diabetes status, and BMI.

	LG2m	GP73	GLAS	FIB‐4	LSM
Age < 65 years	0.75 (0.63–0.86)	0.65 (0.53–0.77)	0.72 (0.61–0.83)	0.88 (0.81–0.94)	0.94 (0.90–0.98)
Age ≥ 65 years	0.50 (0.20–0.81)	0.56 (0.22–0.89)	0.61 (0.28–0.95)	0.61 (0.25–0.98)	0.85 (0.67–1.00)
No diabetes	0.86 (0.70–1.02)	0.84 (0.68–0.99)	0.87 (0.71–1.00)	0.93 (0.87–1.00)	0.94 (0.89–0.99)
Diabetes	0.67 (0.53–0.80)	0.58 (0.45–0.71)	0.65 (0.52–0.77)	0.81 (0.71–0.90)	0.91 (0.84–0.97)
BMI < 30 kg per m^2^	0.71 (0.57–0.84)	0.59 (0.44–0.73)	0.69 (0.55–0.83)	0.84 (0.75–0.94)	0.94 (0.89–0.99)
BMI ≥ 30 kg per m^2^	0.75 (0.58–0.93)	0.71 (0.58–0.93)	0.75 (0.59–0.91)	0.85 (0.75–0.96)	0.92 (0.86–0.98)

Abbreviations: BMI, body mass index; FIB‐4, Fibrosis‐4 index; GLAS, algorithm combining GP73 and LG2m with age and sex; GP73, Golgi protein 73; LG2m, laminin‐γ2 monomer; LSM, liver stiffness measurement.

### 3.5 Role of GLAS in Current Clinical Care Pathway

**FIGURE 4:**
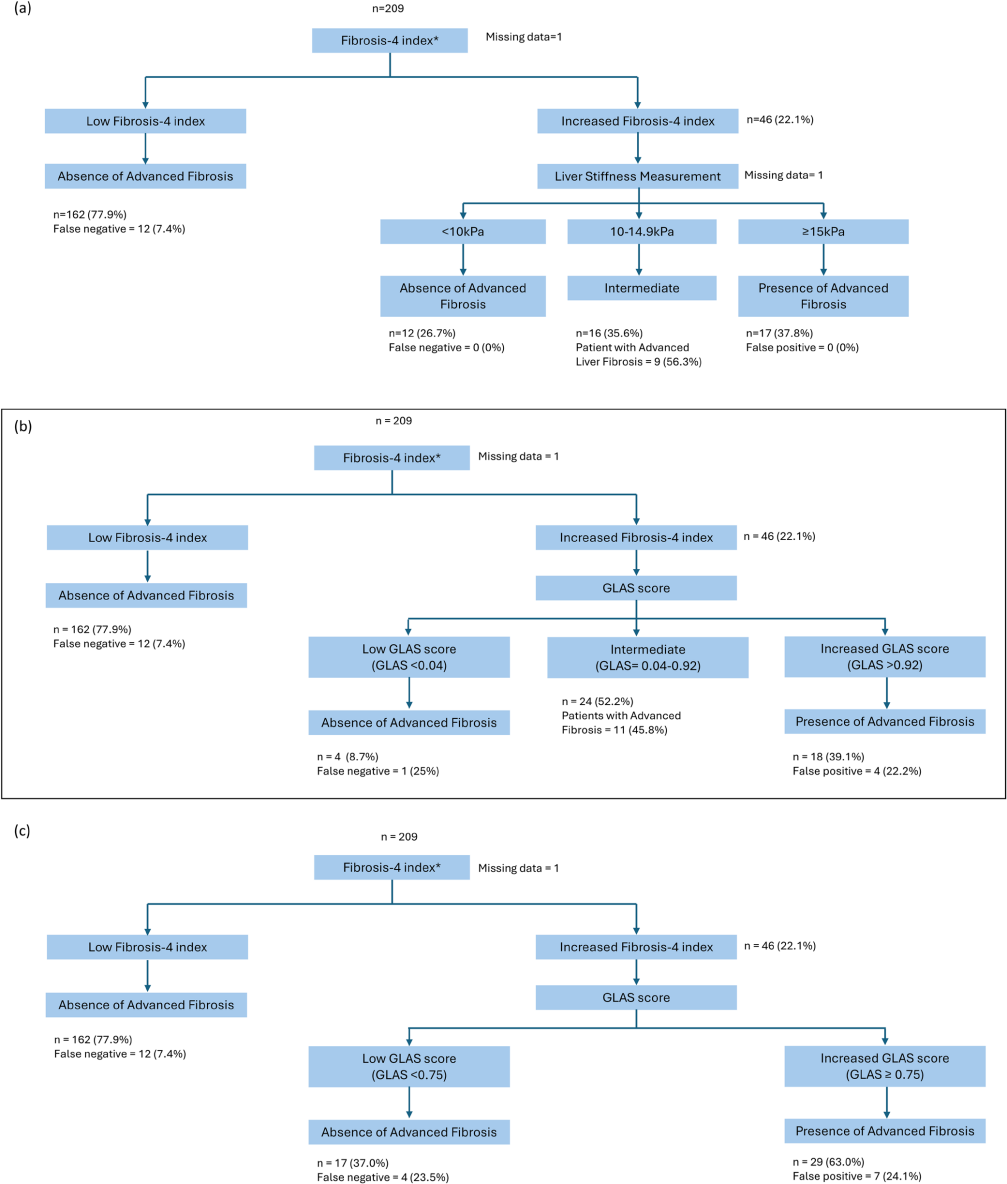
(a) Pathway 1: Diagnosing advanced fibrosis using FIB‐4 followed by LSM, (b) Pathway 2: Diagnosing advanced fibrosis using FIB‐4 followed by the GLAS score using 0.04 (90% sensitivity) and 0.92 (90% specificity) cut‐offs, and (c) Pathway 3: Diagnosing advanced fibrosis using FIB‐4 followed by the GLAS score using optimal cut‐off, 0.75, based on the greatest sum of sensitivity and specificity. *The cut‐off value of < 1.3 was used in patients who are < 65 years old, while the cut‐off value of < 2.0 was used in patients who are ≥ 65 years old, to exclude advanced fibrosis. Overall, in our exploratory analyses on the potential role of GLAS score in the clinical care pathway for the diagnosis of advanced fibrosis, we favor Pathway 3, that is, using the GLAS score as a second test for patients with elevated FIB‐4, when LSM is not available, and also using a single cut‐off for the GLAS score, in view of the reasonable performance and without intermediate results. FIB‐4, Fibrosis‐4 index; GLAS, algorithm combining GP73 and LG2m with age and sex; LSM, liver stiffness measurement.

Exploratory analyses were conducted to evaluate the potential role of GLAS in the clinical care pathway for the diagnosis of advanced fibrosis. The current practice of a two‐step approach (i.e., using FIB‐4 and a subsequent LSM assessment for those with elevated FIB‐4), as recommended by clinical practice guidelines [27, 28, 29, 30, 31, 32], was used as a benchmark (Pathway 1, Figure 4a). Using the low cut‐off, FIB‐4 had a negative predictive value of 92.6% (150/162) and a positive predictive value of 57.8% (26/45) for advanced fibrosis. When LSM was used for patients with elevated FIB‐4, it provided 100% negative predictive value (12/12) and 100% positive predictive value (17/17). Around one third (35.6%, 16/45) of patients with increased FIB‐4 had intermediate LSM of 10–14.9 kPa.

When the GLAS score was explored as an alternative to LSM as a second line test for patients with elevated FIB‐4 (Pathway 2, Figure 4b), it provided a negative predictive value of 75% (3/4) and a positive predictive value of 77.8% (14/18) for diagnosing advanced fibrosis, using the low and high cut‐offs of GLAS of 0.04 and 0.92, corresponding to 90% sensitivity and 90% specificity, respectively. Around half of the patients (52.2%, 24/46) had an intermediate GLAS score of 0.04–0.92. When a single cut‐off of 0.75 (i.e., the optimal cut‐off derived from the ROC analysis) was used for GLAS (Pathway 3, Figure 4c), the negative predictive value was 76.5% (13/17), while the positive predictive value was 75.9% (22/29).

When GLAS was explored as an alternative to FIB‐4 as a first line test using a cut‐off of 0.04 corresponding to 90% sensitivity, the negative predictive value was 83.3% (20/24) while the positive predictive value was 18.4% (33/179). When LSM was used for patients with increased GLAS score, the negative predictive value was 98.3% (115/117) and positive predictive value was 85.7% (18/21). Around one in five patients (22.9%, 41/179) patients were in the intermediate LSM group of 10–14.9 kPa (Pathway 4, Figure S1). Alternatively, when GLAS was used as a third line test at a cut‐off of 0.75 to confirm those with intermediate LSM results (Pathway 5, Figure S2), it resulted in a negative predictive value of 60% (3/5) and positive predictive value of 63.6% (7/11) for the diagnosis of advanced fibrosis. The overall sensitivity, specificity, positive predictive value, negative predictive value and proportion of patients with intermediate results with the different clinical care pathways are shown in Table 3. Overall, in our exploratory analyses on the potential role of GLAS score in the clinical care pathway for the diagnosis of advanced fibrosis, we favor its use as a second test for patients with elevated FIB‐4, when LSM is not available, and also the use of a single cut‐off for the GLAS score that is, Pathway 3, due to the reasonable performance and without intermediate results.

**TABLE 3:** The overall sensitivity, specificity, positive predictive value, negative predictive value, and proportion of patients with intermediate results with 95% confidence interval with the different clinical care pathways.

Pathway	*n*	Cut‐offs	Sensitivity, %	NPV, %	Specificity, %	PPV, %	Intermediate results, %
Pathway 1 (FIB‐4 → LSM)	207	FIB‐4: 1.3 for age < 65, 2 for age ≥ 65; LSM: < 10, 10–14.9, ≥ 15	58.6 (40.7–74.5)	93.1 (88.3–96.0)	100.0 (97.7–100.0)	100.0 (81.6–100.0)	7.7 (4.1–11.4)
Pathway 2 (FIB‐4 → GLAS with dual cut‐offs)	208	FIB‐4: 1.3 for age < 65, 2 for age ≥ 65; GLAS: < 0.04, 0.04–0.92, > 0.92	51.9 (34.0–69.3)	92.2 (87.1–95.4)	97.5 (93.6–99.0)	77.8 (54.8–91.0)	11.5 (7.2–15.9)
Pathway 3 (FIB‐4 → GLAS with single cut‐off)	208	FIB‐4: 1.3 for age < 65, 2 for age ≥ 65; GLAS: < 0.75, ≥ 0.75	57.9 (42.2–73.6)	91.1 (86.9–95.2)	95.9 (92.9–98.9)	75.9 (60.3–91.4)	0 (0.0–1.8)
Pathway 4 (GLAS lower cut‐off → LSM)	203	GLAS: < 0.04, ≥ 0.04; LSM: < 10, 10–14.9, ≥ 15	75.0 (55.1–88.0)	95.7 (91.0–98.0)	97.8 (93.8–99.3)	85.7 (65.4–95.0)	20.2 (14.7–25.7)
Pathway 5 (FIB‐4 → LSM → GLAS with single cut‐off)	207	FIB‐4: 1.3 for age < 65, 2 for age ≥ 65; LSM: < 10, 10–14.9, ≥ 15; GLAS: < 0.75, ≥ 0.75	63.2 (47.3–76.6)	92.2 (87.3–95.3)	97.6 (94.1–99.1)	85.7 (68.5–94.3)	0 (0.0–1.4)

Abbreviations: FIB‐4, fibrosis‐4 score; GLAS, algorithm combining GP73 and LG2m with age and sex; LSM, liver stiffness measurement; NPV, negative predictive value; PPV, positive predictive value.

## 4 Discussion

In our study on a cohort of patients with MASLD who underwent liver biopsy, the GLAS score was found to be a potential new tool for the diagnosis of advanced liver fibrosis with an AUROC of 0.72. However, the results of this study were not as favorable as those of the original study, which reported GLAS scores with an AUROCs of 0.92 and 0.93 in the development and in the validation cohorts, respectively [19]. The superior diagnostic performance in the original study can likely be attributed to the control chosen, whereby cases with fibrosis and cirrhosis were compared with healthy controls. To test the diagnostic goal of detecting advanced fibrosis among patients with MASLD, our study population consisted of patients with MASLD who underwent liver biopsy for suspected MASH (e.g., elevated serum ALT or AST levels ≥ 40 IU/L, significant liver fibrosis based on liver stiffness measurement and/or obese patients with metabolic syndrome) as part of screening for a clinical trial [33]. This highlights the significance of tailoring non‐invasive fibrosis test development and validation to the intended clinical use. Furthermore, cirrhosis was found in 70% and 48% of the patients diagnosed with fibrosis and cirrhosis in the development and validation cohorts of the original study, respectively, while our study population consisted of only 3% of patients with cirrhosis. Both the original study and our research demonstrated that patients with cirrhosis exhibit significantly higher GLAS scores compared to those with less severe fibrosis. Therefore, a study population with a high proportion of cirrhotic patients can be expected to yield a high diagnostic performance for the test. It is also worth noting that patients with MASLD was under‐represented in the original study, contributing to only 15% (39/260) and 2% (6/248) of the patients with fibrosis and cirrhosis in the development and validation cohorts, respectively. Majority of patients in the original study had viral hepatitis or unknown etiology, which contributed to 63% (165/260) and 50% (197/395) of the patients with chronic liver disease in the development and validation cohorts, respectively.

A previous study demonstrated that GP73 can predict NASH in patients with biopsy‐confirmed NAFLD and normal ALT levels, leading to the development of a non‐invasive diagnostic algorithm called G‐NASH, which had an AUROC of 0.85 for the diagnosis of NASH [34]. Similarly, we found GP73 levels to be significantly higher among patients with MASH compared with patients without MASH (Table S1, Figure S3). However, the AUROC for GP73 for the diagnosis of MASH was only 0.62 (95% CI: 0.53–0.70) (Figure S4). In a study on 3044 chronic liver disease patients, serum GP73 showed good diagnostic potential for advanced fibrosis and compensated cirrhosis regardless of etiology. It outperformed APRI and FIB‐4 and showed comparable performance to LSM [10]. Additionally, two other studies [35, 36] demonstrated that GP73 performed well in diagnosing different stages of fibrosis. However, GP73 was found to have lower diagnostic performance for advanced fibrosis in our study compared to previous studies. This is likely due to a greater proportion of patients with early stages of fibrosis in our study population compared with the previous studies, which included more patients with advanced liver fibrosis. Some research suggested that GP73 in combination with LSM has higher diagnostic performance in advanced fibrosis. A study on the diagnosis of NASH and hepatic fibrosis staging in 91 biopsy‐proven NAFLD patients showed that a combination of GP73 and Fibroscan had better diagnostic accuracy than GP73 or Fibroscan alone [37]. In another study on 228 biopsy‐proven MASLD patients, a new algorithm based on GP73, Fibroscan and age (called GFA) was developed with a higher AUROC than FIB‐4, LSM and NFS for the diagnosis of significant liver fibrosis [38].

Current guidelines recommend a two‐step approach for risk stratification [27, 28, 29, 30, 31, 32]. Individuals with MASLD who are identified as having a higher risk of advanced liver fibrosis by a commonly used test (e.g., FIB‐4) are referred for a second line test (e.g., LSM). LSM is easy to use and highly accurate in staging fibrosis, but it is constrained by costs, limited availability and susceptibility to the influence from inflammation and obesity [8, 39]. Therefore, it would be of great value to identify additional blood‐based biomarkers for staging fibrosis. To the best of our knowledge, this is the first study that assessed the application of the GLAS score in a 2‐step approach to diagnose advanced fibrosis. Our study demonstrated that FIB‐4 remains an effective first‐line test due to its reliable performance, particularly in terms of negative predictive value, its affordability, and ease of access. The use of the GLAS score in place of LSM as a second‐line test showed lower overall negative and positive predictive values. However, the GLAS score may be beneficial for patients with elevated FIB‐4 where LSM is unavailable. Notably, when the GLAS score was used as an alternative to LSM as the second‐line test with a single cut‐off (Pathway 3, Figure S3), it yielded overall negative and positive predictive values of 91.1% and 75.9%, respectively, without producing intermediate results that require further workup. This could simplify the diagnostic workflow and reduce the additional burden caused by confirmatory tests for intermediate results. Therefore, among the clinical care pathway that we have evaluated for the GLAS score, we favor the use of the GLAS score as a second test for patients with elevated FIB‐4 when LSM is not available and also the use of a single cut‐off for the GLAS score.

Despite our best efforts, this study has several limitations. First, the sample size was relatively small and limited to patients from a single center. While the distribution of fibrosis stages in this study can be considered as reflective of a population in a specialist clinic, having a larger number of patients with cirrhosis (and a correspondingly larger study population) can provide greater confidence in our observations. Specifically, the subgroup analyses and the analyses on clinical care pathways should be interpreted with caution due to the relatively small sample size (and small number of patients with advanced fibrosis). We also acknowledge that healthy controls included in our study were not matched with the patients with MASLD. However, the healthy controls were not central to the clinical question of identifying advanced fibrosis among patients with MASLD. Their inclusion was to provide additional data of LG2m, GP73 and the GLAS score in persons without fatty liver. Additionally, subjects were patients with MASLD who underwent liver biopsy in a tertiary hospital; thus, they may not represent the broader MASLD patient population. Further validation studies should be conducted in larger, multi‐center cohorts with simulated prevalence of advanced fibrosis to confirm its clinical applicability. Furthermore, the utility of the GLAS score for prognostication and monitoring of disease remains to be explored. In addition, the original GLAS score was developed with limited variables (i.e., GP73, LG2m, age and sex) and may benefit from additional clinical inputs such as diabetes status, platelet count and BMI, further improving the GLAS score in the future. Lastly, GP73 and LG2m were measured using research use only assays. Further work is still needed to optimize these tests for commercial use in routine clinical laboratories as well as to look into its cost effectiveness. Moreover, the formula for the GLAS score is proprietary and was not known to the investigators. These are important factors to consider when judging reproducibility and current clinical applicability. In conclusion, we found serum LG2m level and the GLAS score to have fair accuracy for the diagnosis of advanced fibrosis in patients with MASLD. Although FIB‐4 performed better than the GLAS score, the GLAS score may still have a role as a second line test (where LSM is not available) for patients with elevated FIB‐4 due to its discriminatory value from significantly higher scores in patients with advanced fibrosis.

## Funding

This study was supported by an unrestricted grant from Abbott Laboratories. The company was not involved in the study design, in the collection, analysis, and interpretation of data, in the write up of the report, and in the decision to submit the paper for publication.

## Ethics Statement

This study was approved by the University of Malaya Medical Centre's Medical Research Ethics Committee (MREC ID No.: 2024426‐13664) and conformed to the Declaration of Helsinki.

## Consent

All subjects provided written informed consent.

## Conflicts of Interest

W.K.C. has served as a consultant or advisory board member for Abbott, Abbvie, Boehringer Ingelheim, Novo Nordisk, Roche, and Zuellig Pharma, and a speaker for Abbott, Echosens, Hisky Medical, Novo Nordisk, and Viatris, and received a research grant from Abbott and Roche. The other authors declare no conflicts of interest.

## Supporting information


**Table S1:** Characteristics of patient with and without MASH.
**Figure S1:** Pathway 4: Diagnosing advanced fibrosis using GLAS score followed by LSM.
**Figure S2:** Pathway 5: Diagnosing advanced fibrosis using FIB‐4 followed by LSM and GLAS score.
**Figure S3:** Boxplots showing the serum levels of (a) GP73, and (b) LG2m and (c) the GLAS score in healthy controls and in non‐MASH and MASH patients (*p < 0.05, **p < 0.001).
**Figure S4:** AUROC of GP73, LG2m and the GLAS score for the diagnosis of MASH.

## Data Availability

The data for this study is available upon reasonable request to the corresponding author and with the agreement of the co‐authors. WKC had full access to the data in the study and takes responsibility for the integrity of the data and the accuracy of the data analysis.

## References

[1] Z. M. Younossi , M. Kalligeros , and L. Henry , “Epidemiology of Metabolic Dysfunction‐Associated Steatotic Liver Disease,” Clinical and Molecular Hepatology 31, no. Suppl (2025): S32–S50. 39159948 10.3350/cmh.2024.0431 PMC11925440

[2] M. Ahmed , “Non‐Alcoholic Fatty Liver Disease in 2015,” World Journal of Hepatology 7, no. 11 (2015): 1450–1459. 26085906 10.4254/wjh.v7.i11.1450 PMC4462685

[3] M. Ekstedt , H. Hagström , P. Nasr , et al., “Fibrosis Stage Is the Strongest Predictor for Disease‐Specific Mortality in NAFLD After up to 33 Years of Follow‐Up,” Hepatology 61, no. 5 (2015): 1547–1554. 25125077 10.1002/hep.27368

[4] European Association for the Study of the Liver , European Association for the Study of Diabetes (EASD) , and European Association for the Study of Obesity , “EASL‐EASD‐EASO Clinical Practice Guidelines for the Management of Non‐Alcoholic Fatty Liver Disease,” Obesity Facts 9, no. 2 (2016): 65–90. 27055256 10.1159/000443344 PMC5644799

[5] F. Piccinino , E. Sagnelli , G. Pasquale , et al., “Complications Following Percutaneous Liver Biopsy: A Multicentre Retrospective Study on 68 276 Biopsies,” Journal of Hepatology 2, no. 2 (1986): 165–173. 3958472 10.1016/s0168-8278(86)80075-7

[6] V. Ratziu , F. Charlotte , A. Heurtier , et al., “Sampling Variability of Liver Biopsy in Nonalcoholic Fatty Liver Disease,” Gastroenterology 128, no. 7 (2005): 1898–1906. 15940625 10.1053/j.gastro.2005.03.084

[7] Z. M. Younossi , T. Gramlich , Y. C. Liu , et al., “Nonalcoholic Fatty Liver Disease: Assessment of Variability in Pathologic Interpretations,” Modern Pathology 11, no. 6 (1998): 560–565. 9647594

[8] European Association for Study of Liver; Asociacion Latinoamericana para el Estudio del Higado , “EASL‐ALEH Clinical Practice Guidelines: Non‐Invasive Tests for Evaluation of Liver Disease Severity and Prognosis,” Journal of Hepatology 63, no. 1 (2015): 237–264. 25911335 10.1016/j.jhep.2015.04.006

[9] S. Munro , “Localization of Proteins to the Golgi Apparatus,” Trends in Cell Biology 8, no. 1 (1998): 11–15. 9695801 10.1016/S0962-8924(97)01197-5 PMC7172754

[10] M. Yao , L. Wang , P. S. C. Leung , et al., “The Clinical Significance of GP73 in Immunologically Mediated Chronic Liver Diseases: Experimental Data and Literature Review,” Clinical Reviews in Allergy & Immunology 54 (2018): 282–294. 29256057 10.1007/s12016-017-8655-y

[11] M. Dai , X. Chen , X. Liu , Z. Peng , J. Meng , and S. Dai , “Diagnostic Value of the Combination of Golgi Protein 73 and Alpha‐Fetoprotein in Hepatocellular Carcinoma: A Meta‐Analysis,” PLoS One 10, no. 10 (2015): e0140067. 26441340 10.1371/journal.pone.0140067 PMC4595485

[12] J. Yang , J. Li , W. Dai , et al., “Golgi Protein 73 as a Biomarker for Hepatocellular Carcinoma: A Diagnostic Meta‐Analysis,” Experimental and Therapeutic Medicine 9, no. 4 (2015): 1413–1420. 25780444 10.3892/etm.2015.2231 PMC4353736

[13] F.‐F. Cao , S. Yu , Z. Y. Jiang , and Y. X. Bao , “Diagnostic Accuracy of Golgi Protein 73 in Primary Hepatic Carcinoma Using ELISA: A Systematic Review and Meta‐Analysis,” Clinical Laboratory 60, no. 4 (2014): 587–597. 24779292 10.7754/clin.lab.2013.130312

[14] T. Liu , M. Yao , S. Liu , et al., “Serum Golgi Protein 73 Is Not a Suitable Diagnostic Marker for Hepatocellular Carcinoma,” Oncotarget 8, no. 10 (2017): 16498–16506. 28157705 10.18632/oncotarget.14954 PMC5369980

[15] H. Wei , B. Li , R. Zhang , et al., “Serum GP73, a Marker for Evaluating Progression in Patients With Chronic HBV Infections,” PLoS One 8, no. 2 (2013): e53862. 23418424 10.1371/journal.pone.0053862 PMC3572132

[16] L. Liu , Z. Al‐Dhamin , X. Yuan , et al., “Plasma Golgi Protein 73 Levels Predict Prognosis of HCV‐Related Hepatic Fibrosis,” Histol Histopathol 35, no. 11 (2020): 1309–1318. 33063838 10.14670/HH-18-269

[17] K. Nio , T. Shimakami , T. Terashima , et al., “Serum Laminin γ2 Monomer as a Predictive Biomarker for Hepatocellular Carcinoma in Patients With Chronic Hepatitis B Virus Infection: A Retrospective Cohort Study,” Scientific Reports 14, no. 1 (2024): 25395. 39455696 10.1038/s41598-024-77068-4 PMC11511935

[18] T. Yamashita , N. Koshikawa , T. Shimakami , et al., “Serum Laminin γ2 Monomer as a Diagnostic and Predictive Biomarker for Hepatocellular Carcinoma,” Hepatology 74, no. 2 (2021): 760–775. 33609304 10.1002/hep.31758

[19] P. M. Hemken , X. Qin , L. J. Sokoll , et al., “Validation of the Novel GLAS Algorithm as an Aid in the Detection of Liver Fibrosis and Cirrhosis Based on GP73, LG2m, Age, and Sex,” Clinical Proteomics 20, no. 1 (2023): 53. 38017436 10.1186/s12014-023-09444-7 PMC10683319

[20] N. Chalasani , Z. Younossi , J. E. Lavine , et al., “The Diagnosis and Management of Non‐Alcoholic Fatty Liver Disease: Practice Guideline by the American Gastroenterological Association, American Association for the Study of Liver Diseases, and American College of Gastroenterology,” Gastroenterology 142, no. 7 (2012): 1592–1609. 22656328 10.1053/j.gastro.2012.04.001

[21] M. E. Rinella , J. V. Lazarus , V. Ratziu , et al., “A Multisociety Delphi Consensus Statement on New Fatty Liver Disease Nomenclature,” Hepatology 78 (2023): 1966–1986. 37363821 10.1097/HEP.0000000000000520 PMC10653297

[22] E. Anuurad , K. Shiwaku , A. Nogi , et al., “The New BMI Criteria for Asians by the Regional Office for the Western Pacific Region of WHO Are Suitable for Screening of Overweight to Prevent Metabolic Syndrome in Elder Japanese Workers,” Journal of Occupational Health 45, no. 6 (2003): 335–343. 14676412 10.1539/joh.45.335

[23] K. G. M. Alberti , P. Zimmet , and J. Shaw , “The Metabolic Syndrome—A New Worldwide Definition,” Lancet 366, no. 9491 (2005): 1059–1062. 16182882 10.1016/S0140-6736(05)67402-8

[24] D. E. Kleiner , E. M. Brunt , M. van Natta , et al., “Design and Validation of a Histological Scoring System for Nonalcoholic Fatty Liver Disease,” Hepatology 41, no. 6 (2005): 1313–1321. 15915461 10.1002/hep.20701

[25] S. McPherson , T. Hardy , J. F. Dufour , et al., “Age as a Confounding Factor for the Accurate Non‐Invasive Diagnosis of Advanced NAFLD Fibrosis,” Official Journal of the American College of Gastroenterology| ACG 112, no. 5 (2017): 740–751. 10.1038/ajg.2016.453 PMC5418560 27725647

[26] J. Boursier , J. P. Zarski , V. de Ledinghen , et al., “Determination of Reliability Criteria for Liver Stiffness Evaluation by Transient Elastography,” Hepatology 57, no. 3 (2013): 1182–1191. 22899556 10.1002/hep.25993

[27] W. K. Chan , S. Treeprasertsuk , G. B. Goh , et al., “Optimizing Use of Nonalcoholic Fatty Liver Disease Fibrosis Score, Fibrosis‐4 Score, and Liver Stiffness Measurement to Identify Patients With Advanced Fibrosis,” Clinical Gastroenterology and Hepatology 17, no. 12 (2019): 2570–2580. 30876959 10.1016/j.cgh.2019.03.006

[28] European Association for the Study of the L , European Association for the Study of D , and European Association for the Study of O , “EASL‐EASD‐EASO Clinical Practice Guidelines on the Management of Metabolic Dysfunction‐Associated Steatotic Liver Disease (MASLD),” Journal of Hepatology 81, no. 3 (2024): 492–542. 38851997 10.1016/j.jhep.2024.04.031

[29] M. E. Rinella , B. A. Neuschwander‐Tetri , M. S. Siddiqui , et al., “AASLD Practice Guidance on the Clinical Assessment and Management of Nonalcoholic Fatty Liver Disease,” Hepatology 77, no. 5 (2023): 1797–1835. 36727674 10.1097/HEP.0000000000000323 PMC10735173

[30] M. Eslam , J. G. Fan , M. L. Yu , et al., “The Asian Pacific Association for the Study of the Liver Clinical Practice Guidelines for the Diagnosis and Management of Metabolic Dysfunction‐Associated Fatty Liver Disease,” Hepatology International 19, no. 2 (2025): 261–301. 40016576 10.1007/s12072-024-10774-3

[31] W. K. Chan , S. S. Tan , S. P. Chan , et al., “Malaysian Society of Gastroenterology and Hepatology Consensus Statement on Metabolic Dysfunction‐Associated Fatty Liver Disease,” Journal of Gastroenterology and Hepatology 37, no. 5 (2022): 795–811. 35080048 10.1111/jgh.15787 PMC9303255

[32] W. K. Chan , K. H. Chuah , R. B. Rajaram , L. L. Lim , J. Ratnasingam , and S. R. Vethakkan , “Metabolic Dysfunction‐Associated Steatotic Liver Disease (MASLD): A State‐of‐the‐Art Review,” Journal of Obesity & Metabolic Syndrome 32, no. 3 (2023): 197–213. 37700494 10.7570/jomes23052 PMC10583766

[33] C. W. Kheong , N. R. N. Mustapha , and S. Mahadeva , “A Randomized Trial of Silymarin for the Treatment of Nonalcoholic Steatohepatitis,” Clinical Gastroenterology and Hepatology 15, no. 12 (2017): 1940–1949. 28419855 10.1016/j.cgh.2017.04.016

[34] K. I. Zheng , W. Y. Liu , X. Y. Pan , et al., “Combined and Sequential Non‐Invasive Approach to Diagnosing Non‐Alcoholic Steatohepatitis in Patients With Non‐Alcoholic Fatty Liver Disease and Persistently Normal Alanine Aminotransferase Levels,” BMJ Open Diabetes Research & Care 8, no. 1 (2020): e001174. 10.1136/bmjdrc-2020-001174 PMC7059499 32139603

[35] S. Peng , Q. Wu , X. Huang , et al., “Diagnostic Value of Serum Golgi Protein 73 for Liver Fibrosis: A Systematic Review and Meta‐Analysis,” Digestive Diseases 41, no. 4 (2023): 622–631. 36812901 10.1159/000529815

[36] C. Hui‐Ling , H. Kang‐Ming , Z. Yu , et al., “The Potential Value of Serum GP73 in the Ancillary Diagnosis and Grading of Liver Cirrhosis,” Scandinavian Journal of Clinical and Laboratory Investigation 83, no. 2 (2023): 95–102. 36786815 10.1080/00365513.2023.2175238

[37] Y. Li , Y. Yang , Y. Li , et al., “Use of GP73 in the Diagnosis of Non‐Alcoholic Steatohepatitis and the Staging of Hepatic Fibrosis,” Journal of International Medical Research 49, no. 11 (2021): 3000605211055378. 34772312 10.1177/03000605211055378 PMC8593324

[38] S. Hong , Z. Liu , P. Li , J. Zhang , and H. Wei , “Golgi Protein 73: Charting New Territories in Diagnosing Significant Fibrosis in MASLD: A Prospective Cross‐Sectional Study,” Frontiers in Endocrinology 15 (2025): 1506953. 39872312 10.3389/fendo.2024.1506953 PMC11769827

[39] L. Castéra , J. Foucher , P. H. Bernard , et al., “Pitfalls of Liver Stiffness Measurement: A 5‐Year Prospective Study of 13,369 Examinations,” Hepatology 51, no. 3 (2010): 828–835. 20063276 10.1002/hep.23425

