# Supplementary material for: Golgi Protein 73 and Laminin‐γ2 Monomer‐Based Score for the Diagnosis of Advanced Liver Fibrosis in Patients With Metabolic Dysfunction‐Associated Steatotic Liver Disease
Source: JGH Open. 2026 Aug 2;10(8):e70455. doi: 10.1002/jgh3.70455 (PMC13429801; doi:10.1002/jgh3.70455)
Supplement: Supplementary file 1 — Table S1: Characteristics of patient with and without MASH. Figure S1: Pathway 4: Diagnosing advanced fibrosis using GLAS score followed by LSM. Figure S2: Pathway 5: Diagnosing advanced fibrosis using FIB‐4 followed by LSM and GLAS score. Figure S3: Boxplots showing the serum levels of (a) GP73, and (b) LG2m and (c) the GLAS score in healthy controls and in non‐MASH and MASH patients (*p < 0.05, **p < 0.001). Figure S4: AUROC of GP73, LG2m and the GLAS score for the diagnosis of MASH. [file JGH3-10-e70455-s001.docx]

**Golgi protein 73 and laminin‐γ2 monomer-based score for the diagnosis of advanced liver fibrosis in patients with metabolic dysfunction-associated steatotic liver disease: supplementary material**

Xin-Tong Ng¹^, *^, Jin-Ying Teh¹^, *^, Pavai Sthaneshwar^2^, Izzatul Aliaa Badaruddin^3^, Pavitratha Puispanathan^4^, Nik Raihan Nik Mustapha^5^, Sanjiv Mahadeva^1^, Wah-Kheong Chan¹

*^1^Gastroenterology and Hepatology Unit, Department of Medicine, Faculty of Medicine, University of Malaya, Kuala Lumpur, Malaysia*

*^2^Clinical Diagnostic Laboratory, Department of Pathology, Faculty of Medicine, University of Malaya, Kuala Lumpur, Malaysia*

*^3^Medical Diagnostic Laboratory, Department of Pathology, Faculty of Medicine, Universiti Kebangsaan Malaysia, Bangi, Selangor, Malaysia*

*^4^Department of Pathology, Hospital Pulau Pinang, Pulau Pinang, Malaysia*

*^5^Department of Pathology, Hospital Sultanah Bahiyah, Alor Setar, Kedah, Malaysia*

*Xin-Tong Ng and Jin-Ying Teh are co-first authors for this work

Corresponding author:

Dr Wah-Kheong Chan

Gastroenterology and Hepatology Unit, Department of Medicine, Faculty of Medicine, University of Malaya, 50603 Kuala Lumpur, Malaysia

Telephone No.: +60379492965

E-mail: [wahkheong2003@hotmail.com](mailto:wahkheong2003@hotmail.com)

**Supplementary Table S1** Characteristics of patient with and without MASH

|  | Overall, n = 209 | Non-MASH, n = 60 | MASH, n = 149 | p-value* |
| --- | --- | --- | --- | --- |
| Age, years | 52.0 (42.0 – 58.0) | 47.0 (38.3 – 55.8) | 53.0 (44.0 – 59.5) | 0.023 |
| Male, % | 53.1 | 63.3 | 49.0 | 0.060 |
| Weight, kg | 78.1 ± 14.3 | 74.9 ± 12.8 | 79.4 ± 14.8 | 0.037 |
| Obesity, % | 86.1 | 80.0 | 88.6 | 0.104 |
| BMI, kg per m^2^ | 29.5 (26.7 – 32.2) | 27.6 (25.5 – 30.7) | 30.3 (27.6 – 33.0) | 0.001 |
| Waist circumference, cm | 98 ± 10 | 95 ± 9 | 100 ± 10 | 0.005 |
| Central Obesity, % | 94.7 | 85.0 | 98.7 | <0.001 |
| Diabetes, % | 52.6 | 35.0 | 59.7 | 0.001 |
| Hypertension, % | 59.3 | 41.7 | 66.4 | <0.001 |
| Dyslipidemia, % | 74.2 | 68.3 | 76.5 | 0.222 |
| Ischemic heart disease, % | 2.9 | 3.3 | 2.7 | 0.799 |
| CAP, dB/m | 327 (295 – 347) | 328 (283 – 343) | 327 (297 – 350) | 0.300 |
| IQR for CAP, dB/m | 7 (5 – 10) | 7 (6 – 9) | 7 (5 – 10) | 0.772 |
| E, kPa | 7.7 (5.9 – 11.4) | 6.2 (4.8 – 7.3) | 8.9 (6.7 – 12.2) | <0.001 |
| IQR/Median for E, % | 12.5 (9.0 – 18.0) | 13.5 (8.0 – 19.8) | 12.0 (9.0 – 17.0) | 0.955 |
| FBS, mmol/L | 5.8 (5.1 – 7.1) | 5.6 (5.0 – 6.3) | 6.0 (5.2 – 7.2) | 0.038 |
| Serum Insulin level, mIU/L | 23.1 (16.4 – 39.2) | 16.8 (12.3 – 25.9) | 26.1 (18.3 – 41.2) | <0.001 |
| HbA1C, % | 6.1 (5.6 – 7.2) | 5.7 (5.3 – 6.2) | 6.5 (5.7 – 7.5) | <0.001 |
| Albumin, g/L | 43 (41 – 45) | 44 (41 – 46) | 43 (41 – 45) | 0.089 |
| Bilirubin, umol/L | 10 (8 – 15) | 10 (7 – 15) | 10 (8 – 15) | 0.567 |
| ALT, IU/L | 64 (43 – 102) | 48 (35 – 72) | 73 (48 – 109) | <0.001 |
| AST, IU/L | 39 (28 – 61) | 29 (23 – 38) | 48 (32 – 69) | <0.001 |
| GGT, IU/L | 77 (41 – 116) | 45 (31 – 89) | 82 (49 – 127) | <0.001 |
| Platelet, 10^9/L | 274 ± 68 | 290 ± 59 | 267 ± 70 | 0.030 |
| Triglyceride, mmol/L | 1.60 (1.30 – 2.00) | 1.60 (1.30 – 2.00) | 1.60 (1.30 – 2.00) | 0.994 |
| Total cholesterol, mmol/L | 4.80 (4.20 – 5.60) | 5.10 (4.40 – 5.70) | 4.70 (4.15 – 5.55) | 0.045 |
| HDL, mmol/L | 1.16 (0.99 – 1.33) | 1.20 (1.00 – 1.37) | 1.13 (0.98 – 1.30) | 0.216 |
| LDL, mmol/L | 2.90 (2.35 – 3.59) | 3.05 (2.48 – 3.57) | 2.77 (2.25 – 3.63) | 0.086 |

*p-value between groups, i.e., NASH and non-NASH, were calculated using t-test or Mann-Whitney test, where appropriate, for continuous variables, and chi-square test or Fisher exact test, where appropriate, for categorical variables.

Note: MASH was defined as the presence of steatosis, lobular inflammation and ballooning with or without fibrosis.

*BMI, body mass index; CAP, controlled attenuation parameter; IQR, interquartile range; E, liver stiffness measurement; FBS, fasting blood sugar; HbA1C, glycated haemoglobin; ALT, alanine aminotransferase, AST, aspartate aminotransferase; GGT, gamma-glutamyl transferase; HDL, high-density lipoprotein cholesterol; LDL, low-density lipoprotein cholesterol.*

**Supplementary Figure S1** Pathway 4: Diagnosing advanced fibrosis using GLAS score followed by LSM


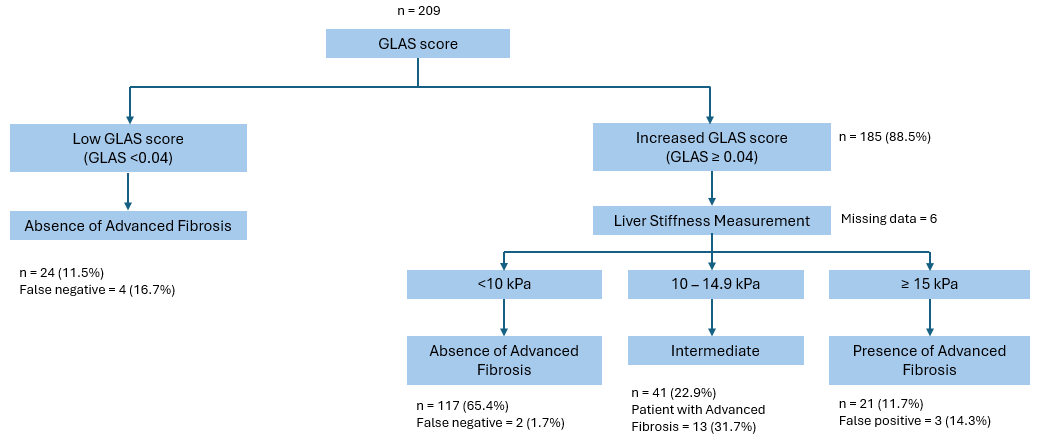


Note: The 0.04 cut-off for the GLAS score had 90% sensitivity for identifying advanced fibrosis.

*GLAS, algorithm combining GP73 and LG2m with age and sex*

**Supplementary Figure S2** Pathway 5: Diagnosing advanced fibrosis using FIB-4 followed by LSM and GLAS score


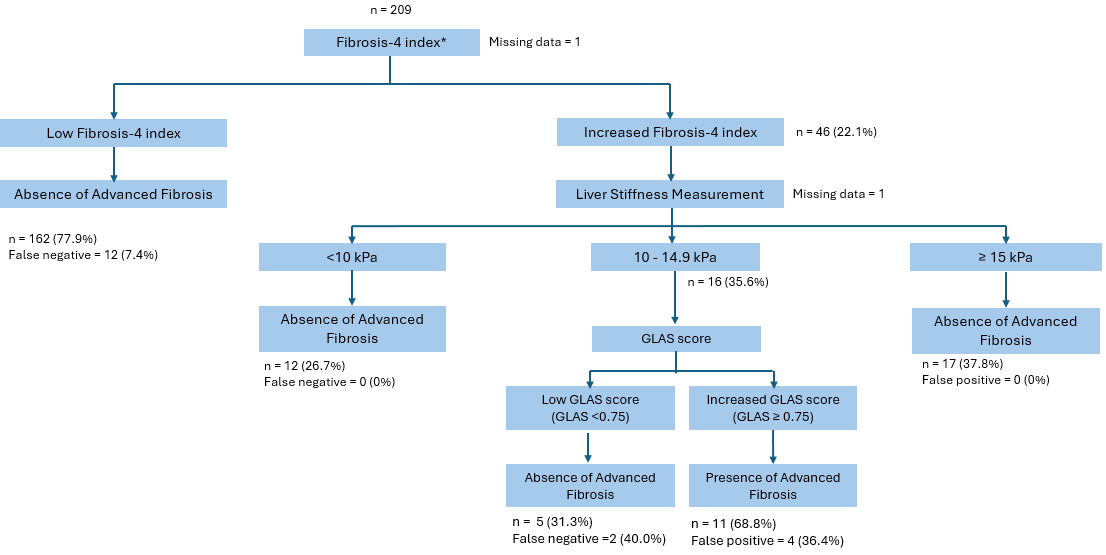


*The cut-off value of <1.3 was used in patients who are <65 years old, while the cut-off value of <2.0 was used in patients who are ≥65 years old, to exclude advanced fibrosis.

Note: The 0.75 cut-off for the GLAS score was the optimal cut-off based on greatest sum of sensitivity and specificity.

*GLAS, algorithm combining GP73 and LG2m with age and sex*

**Supplementary Figure S3** Boxplots showing the serum levels of (a) GP73, and (b) LG2m and (c) the GLAS score in healthy controls and in non-MASH and MASH patients (*p<0.05, **p<0.001)


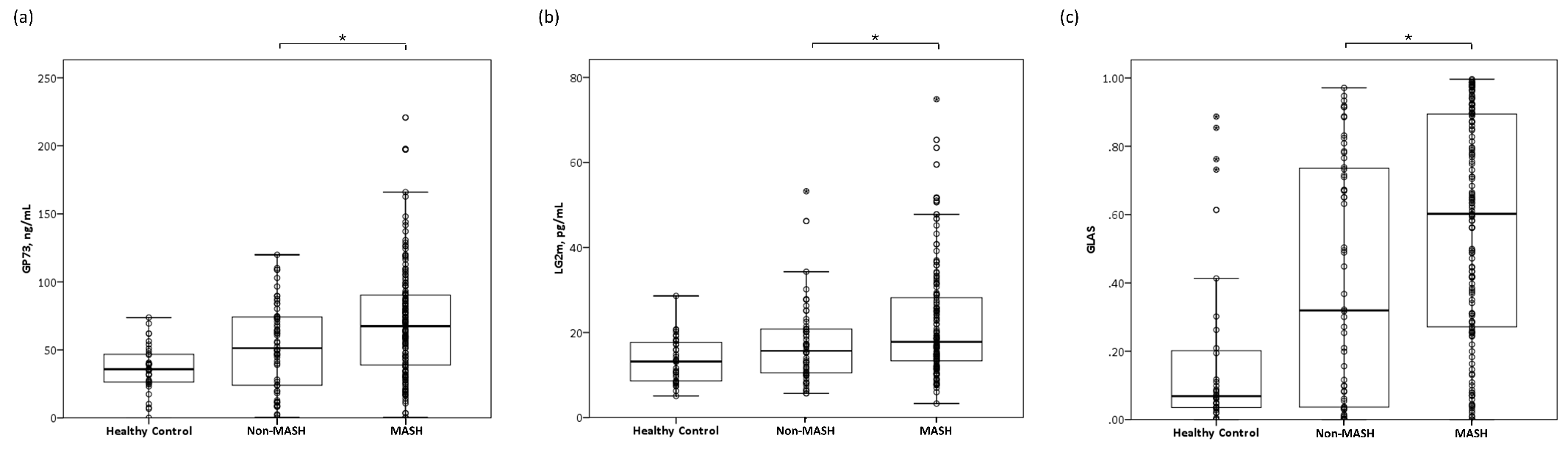


Note: The serum levels of GP73 and LG2m and the GLAS scores were significantly higher in patients with MASH compared to those without MASH (p=0.009, p=0.014, and p=0.002, respectively).

*GLAS, algorithm combining GP73 and LG2m with age and sex; GP73, Golgi protein 73; LG2m, laminin‐γ2 monomer; MASH, metabolic dysfunction-associated steatohepatitis*

**Supplementary Figure S4** AUROC of GP73, LG2m and the GLAS score for the diagnosis of MASH


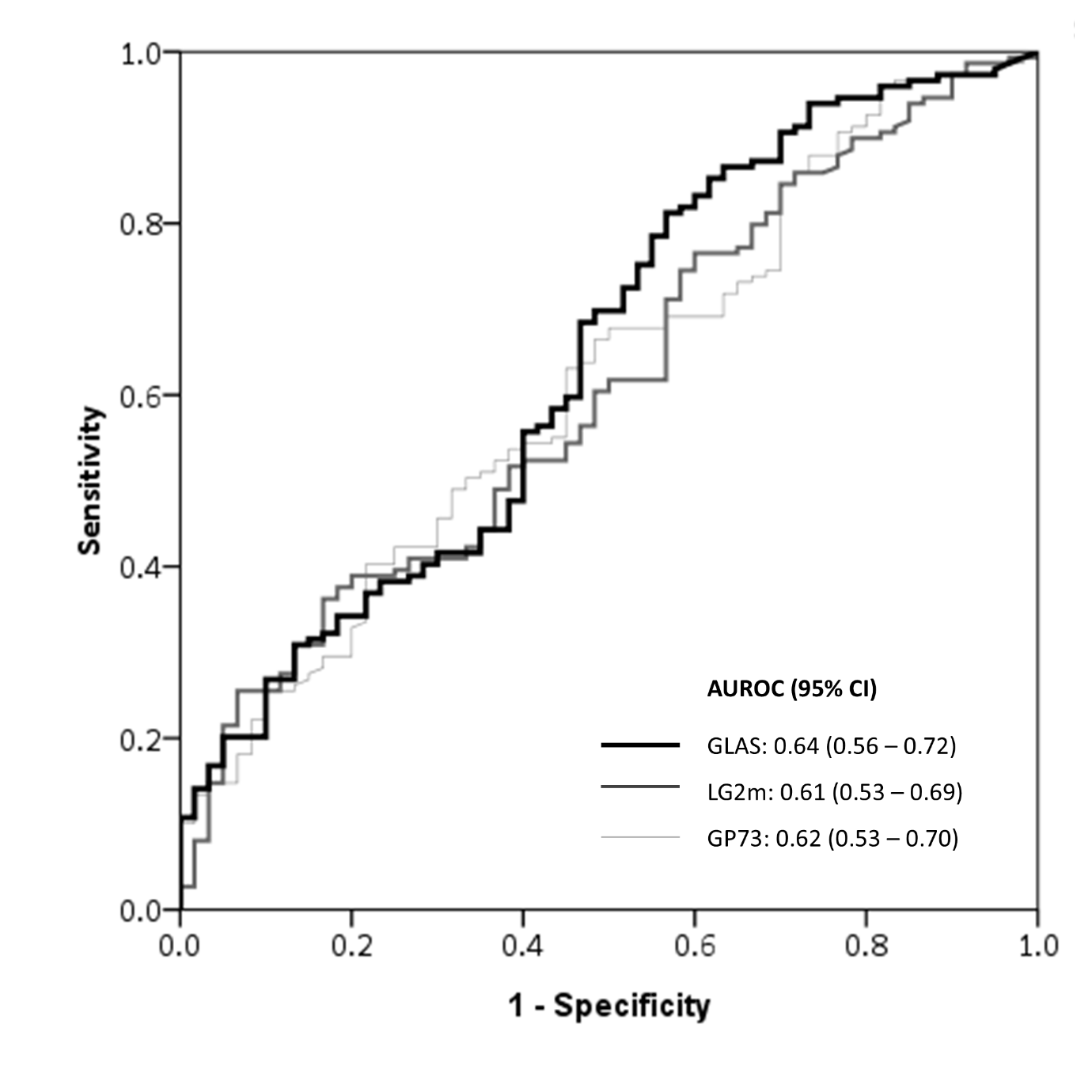


Note: AUROC was interpreted as follows: 0.90–1.00 = excellent, 0.80–0.90 = good, 0.70–0.80 = fair, < 0.70 = poor.

*GLAS, algorithm combining GP73 and LG2m with age and sex; GP73, Golgi protein 73; LG2m, laminin‐γ2 monomer*
